# Phase II Study of S-1 Monotherapy as a First-line, Combination Therapy of S-1 plus Cisplatin as a Second-line, and Weekly Paclitaxel Monotherapy as a Third-line Therapy in Patients with Advanced Gastric Carcinoma: Phase II Study of S-1, S-1 plus Cisplatin, and Weekly Paclitaxel in Patients with Advanced Gastric Carcinoma

**DOI:** 10.4137/cmo.s610

**Published:** 2008-04-28

**Authors:** Yasushi Rino, Norio Yukawa, Nobuyuki Wada, Makoto Suzuki, Hitoshi Murakami, Takanobu Yamada, Hirotaka Nakayama, Naoto Yamamoto, Tsutomu Sato, Roppei Yamada, Takashi Ohshima, Munetaka Masuda, Toshio Imada

**Affiliations:** 1 Department of Surgery, School of Medicine, Yokohama City University; 2 Department of Gastroenterological Center, Medical Center, Yokohama City University; 3 Hospital of Yokohama City University

**Keywords:** advanced gastric cancer, recurrent gastric cancer, S-1, cisplatin, paclitaxel, phase II study, first-line chemotherapy, second-line chemotherapy, third-line chemotherapy

## Abstract

**Background:**

We conducted a pilot phase II study to evaluate the efficacy and safety of S-1 as a first-line, S-1 plus cisplatin as a second-line, and weekly paclitaxel as a third-line therapy for advanced gastric cancer.

**Patients and methods:**

Between 2002 and 2005, 19 patients were enrolled in this study. Chemotherapy consisted of either 60 mg/m^2^ of S-1 for 4 weeks at 6 weeks interval, a combination of 60 mg/m^2^ S-1 for 3 weeks and 60 mg/m^2^ cisplatin on day 8 at 5 weeks interval, or 60 mg/m^2^ paclitaxel at day 1, 8, 15, at 4 weeks interval. The regimen was repeated until the occurrence of unacceptable toxicities, disease progression, or patient refusal. The primary end point was the overall survival.

**Results:**

The response rates were 33.3%, 12.5%, and 0% after the first, second, and third line chemotherapy, respectively. The mean overall survival time was 994 days. The median survival time could not be calculated because 12 out of 19 patients were still alive when the study was concluded. Regarding hematological toxicity, the major adverse effect was leukopenia, which reached grades 3–4 in all lines of chemotherapy investigated. In addition, regarding non-hematological toxicities, the major adverse effect was anorexia, which reached grade 3–4 in the second line chemotherapy, and no deaths were attributable to the adverse effects of the drugs.

**Conclusion:**

This sequential therapy was an effective treatment for advanced gastric cancer with acceptable toxic side-effects. We considered this sequential therapy to be effective because of the smooth switch to the next regimen.

## Introduction

Recently, several clinical trials have demonstrated the efficacy of certain chemotherapeutic agents against gastric cancer (1,2). Oral fluorouracil antitumor drugs were first developed in 1971 in Japan, leading to the establishment of UFT (3). S-1 is a novel oral fluorouracil antitumor drug that contains a combination of 3 pharmacological agents: tegafur (FT), a 5-Fluorouracil (5-FU) prodrug, 5-chloro-2, 4-dihydroxypyridine (CDHP), which inhibits the activity of dihydropyrimidine dehydrogenase (DPD) and potassium oxonate (Oxo), which reduces the gastrointestinal toxicity of 5-FU. A phase II study of S-1 showed a 45% response rate to the drug and a median survival time of 275 days (4–10). This drug has gradually been accepted as the front-line chemotherapeutic agent in Japan for the treatment of gastric cancer cases that were unresectable, resected but not cured, or recurrent. Unfortunately, some gastric cancers do not respond to this agent. Therefore, in cases who S-1 therapy failed, we administered a second-line regimen, using or adding other agents, namely, cisplatin, paclitaxel, docetaxel and irinotecan. When a patient administered S-1 had progressive disease, after treatment with cisplatin, paclitaxel, docetaxel or irinotecan without cross-tolerance of S-1 resulted in good outcome.

However, we could not find any reports investigating a third-line chemotherapy, administered drugs without cross-tolerance of prior chemotherapy. As such, we decided to carry out a single administration of S-1, and as the second-line chemotherapy in which cisplatin is additionally administered, a regimen of S-1 plus cisplatin. The regimen of S-1 plus cisplatin had a high response rate of over 70%, and it was also reported that side-effects at grade 3 or above accounted for less than 25% (11), so it was concluded that curative effects can be expected even after the administration of S-1. However, there is no evidence that clearly shows whether the regimen of S-1 plus cisplatin after S-1 failure is effective or not. Next, as the third-line chemotherapy, we decided to provide weekly paclitaxel therapy, which has no cross-tolerance.

In this study, we evaluated S-1, S-1 plus cisplatin, and paclitaxel as chemotherapy for advanced gastric cancer, by measuring the objective response rate, the overall survival and the safety profile, for use as a first-, second- and third-line chemotherapeutic regimens.

## Patients and Methods

### Patient eligibility

A series of 19 patients were enrolled in this study between June 2002 and December 2005. To be eligible for inclusion, the patients had to have histologically or cytologically confirmed gastric adenocarcinoma that was either unresectable (n = 3), palliatively resected (n = 10) or recurrent (n = 6) (Table 1). The patients had to have no prior chemotherapy received and were able to take S-1 orally. Recurrent patients were allowed to be included if at least 3 months has elapsed after the last post operative adjuvant chemotherapy. S-1 was administered daily for two 4-week periods, separated by a 2-week interval for the first-line chemotherapy, after an informed consent from the patient has been obtained. A dose reduction was not allowed; however, the treatment schedule was changed in some cases to 5 consecutive days of treatment followed by 2 days of rest per week. This treatment schedule was repeated for 4 weeks, followed by 2 weeks of rest. The regimen was changed if more than a grade 3 toxicity, progressed disease and escalated tumor marker were shown. However, if grade 3–4 toxicity was observed after the first-line chemotherapy, then a second-line chemotherapy was administered after the patient recovered from the toxicity. If the second-line chemotherapy was refused by the patient who originally received S-1, then weekly paclitaxel was given as the second-line chemotherapy. The patients were also required to meet the following criteria: age #75 years, amenable to oral administration of drugs, a Karnofsky performance score of 60, a life expectancy of at least 3 months, and an adequate hematological status (defined as having a total leukocyte count >3,500/mm^3^, neutrophil count > 1,500/mm^3^, platelet count >100,000/mm^3^, serum creatinine <1.5 mg/dl, total serum bilirubin <1.5 mg/dl, aspartate aminotransferase(AST)and alanine aminotransferase (ALT) levels less than 2 times the upper limit of the normal range). Patients were excluded from the study if they had any other current or prior malignancies, active uncontrolled infections or other diseases, or a neurological or mental disease that prevented adequate comprehension of information. The pretreatment evaluation consisted of a complete history and physical examination, blood count, serum biochemistry, and computed tomography (CT) of the chest and abdomen. All patients gave their informed consent before the initiation of treatment.

### Study design (Fig. 1)

#### First line chemotherapy

S-1 was administered orally twice daily after breakfast or dinner, at 80 mg/m^2^/day for 4 weeks, followed by 2 weeks of rest. During the course of the treatment, the patients were evaluated the complete blood count (CBC), biochemical and physical examinations on every 2 weeks, and for the presence of tumor markers (CEA, CA19-9, STn and SLX) on every 4 weeks. The treatment response was then evaluated by computed tomography (CT) on every 2 months.

#### Second line chemotherapy

S-1 was administered at the same dosage as the first line chemotherapy. S-1 was administered for 3 weeks followed by 2 weeks of rest. On day 8, S1 was combined with cisplatin at 60 mg/m^2^. The patients were pre-medicated with 8 mg dexamethasone and 10 mg azasetron hydrochrolide diluted in 50 ml of saline, given intravenously 30 minutes prior to treatment. Chemotherapy was then administered by intravenous infusion and it consisted of 60 mg/m^2^ cisplatin administered over 120 minutes. During the course of the treatment, the patients were evaluated CBC, biochemical and physical examinations on every 2–3 weeks, and on every 4 weeks, for tumor markers (CEA, CA19-9, STn and SLX). The treatment response was evaluated on every 2 months by CT.

#### Third line chemotherapy

Paclitaxel was administered at 60 mg/m^2^ on day 1, 8, and 15 of a 4-week treatment cycle. Prior to the paclitaxel administration, the patients were given 50 mg diphenhydramine orally and 20 mg dexamethasone intravenously with 50 ml saline water over 30 min, 50 mg ranitidine hydrochloride intravenously with 50 ml saline water over 30 min, 10 mg azasetron hydrochrolid intravenously with 50 ml saline water over 30 min. Paclitaxel was administered by intravenous infusion and it consisted of 60 mg/m^2^ over 60 minutes.

All patients were admitted to the hospital for the first intravenous treatment of the second and third line chemotherapy. The succeeding intravenous treatments were performed on an out-patient basis.

If hematological or non-hematological toxicities of grade 3 or higher occurred, or if patients requested that the treatment be stopped.

### Study evaluations

All responses were assessed by physical examination, direct visualization, examination of the upper gastrointestinal tract after a barium meal, gastro-fibroscopy and CT. A tumor evaluation was carried out every two months, according to the World Health Organization criteria, and the responses were confirmed by radiography within 2 weeks. A complete response (CR) was defined as a remission of all diseases for a minimum of 4 weeks. A partial response (PR) was defined as a >50% reduction in the product of the perpendicular diameters of the indicator lesions, without the appearance of new lesions. Progressive disease (PD) was defined as an enlargement of >25% in an indicator lesion or the development of new lesions, and no change (NC) was defined as a failure to meet the criteria for either the response or progression. All adverse events were graded using the National Cancer Institute Common Toxicity Criteria (NCI-CTC) at each treatment cycle. In the event of toxicity, chemotherapy was postponed until the symptoms had resolved.

### Survival analysis

Because the few patients had measurable lesion, the primary end point of this study was the overall survival (OS). The Kaplan-Meier method was used to calculate the survival rate. The difference between the curves was assessed using the log-rank test. Results with probability (p) values < = 0.05 were considered to be statistically significant. A statistical calculation was conducted using the Dr. SPSS II for Windows software program.

## Results

### Patient characteristics

The demographic features of the 19 patients enrolled in this study are shown in Table I. All patients were assessed for response and toxicity. The median patient age was 61.3 years (range: 38–75 years); 12 patients were male (63.2%) and 7 were female (36.8%), with all patients being in good general condition (Karnofsky performance status: 80–100). All patients had histologically confirmed adenocarcinoma, with 6 differentiated, and 13 undifferentiated adenocarcinomas. The most frequently observed sites of tumors were the peritoneal dissemination in 14 patients, the lymph nodes metastasis in 5 patients, followed by the primary tumor site in 3 patients and liver metastases in 2 patients.

The first-line was performed in 19 cases, and 6.2 courses of administration, on average, could be performed (range: 0–20). The second-line was performed in 13 cases, with 2.8 courses on average performed (range: 1–7). The third-line was performed in 13 cases, with 3.8 courses on average (range: 0–14). The cases with 0 courses in the first-line and third-line were the result of refusal by the patients due to side-effects as well as rapidly increased lesions. The fourth-line and subsequent treatments were performed in 8 cases. None of the patients underwent subsequent surgery.

### Efficacy

There were assessable lesions in 9 cases within the first-line, 8 cases in the second-line, and 4 cases in the third-line. In the first-line, 1 case of CR and 2 cases of PR were found, and in the second-line, there was 1 case of PR, but in the third-line neither PR nor CR were found. The response rates (RR) were 33.3%, 12.5%, and 0% for each therapy. As for response regions, in CR for the first-line, there was lymph node metastasis, and in PR, there were liver metastasis, peritoneal dissemination, and primary tumors. There was liver metastasis in PR for the second-line.

### Survival

**Figure 2:**
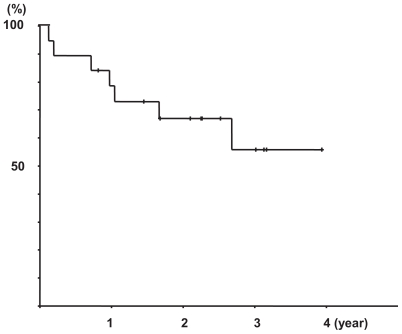
Over all Survival curve (n = 19) The mean survival time was 994.0 ± 127.1 days.

**Figure 3:**
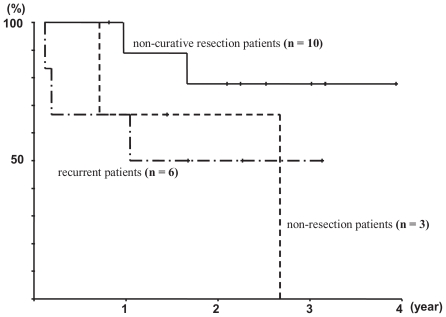
Survival curves of every primary treatments In the non-curative resection patients (n = 10), the mean survival time was 1207.4 ± 132.1 days and the median survival time (MST) was not calculated. In the non-resection patients (n = 3), the mean survival time was 727.7 ± 271.7 days, and the MST was 522 days. In the recurrent patients (n = 6), the mean survival time was 644.5 ± 201.3 days and the MST was 376 days. There were no significant differences between the non-curative resection, non- resection and recurrent patients.

The mean follow-up time was 726 days with a range of 44 to 1461 days. The mean time to progression was 314.3 days with a range of 33 to 845 days. The median survival time (MST) for patients over the whole course of their treatment was not calculated because 12 out of 19 patients were still alive. The mean survival time was 994 days (Fig. 2). In the non-curative resection patients (n = 10), the mean survival time was 1207.4 days and the MST was not calculated because the 8 patients were still alive. In the non-resection patients (n = 3), the mean survival time was 727.7 days, 2 patients died, and the MST was 522 days. In the recurrent patients (n = 6), the mean survival time was 644.5 days, 3 patients died, and the MST was 376 days. There were no significant differences between the non-curative resection, non- resection, and recurrent patients (Fig. 3).

### Toxicity

Regarding hematological toxicity, leucopenia, which is a side-effect with at least grade 3 severity, was most frequently found, followed by anemia. In the first-line, 1 case of leucopenia and 1 case of neutropenia (10.5%) were found; in the second-line, 3 cases of leucopenia and 2 cases of anemia (23.1%); and in the third-line, 2 cases of leucopenia and 1 case of anemia (23.1%). As for non-hematological toxicity, a loss of appetite was most frequently found. In the first-line, 1 case of stomatitis, 1 case of lachrymation, and 1 case of numbness in limbs (10.5%); in the second-line, 1 case of nausea, 4 cases with a loss of appetite, 2 cases of diarrhea, 1 case of fatigue, and 1 case of abdominal pain (46.2%); and in the third-line, not a single case was found. (Table 2)

## Discussion

S-1 has been the most widely used anti-cancer drug for advanced or recurrent gastric cancer in Japan. S-1, a newly developed oral tegafur compound, contains CDHP, which transiently, but strongly, inhibits DPD. The presence of this enzyme in the formulation allows the plasma concentration of 5-FU to be maintained at a high level for 8 h, giving a high response rate for gastric cancer. Another phase II study has shown a response rate to S-1 of 45% and a median survival time of 275 days. In addition, S-1 treatment can be administered on an outpatient basis, which is a striking difference from other intensive chemotherapies, including regimens with low-dose 5-fluorouracil and cisplatin (12,13) and MTX/5FU (14). The high response rate thereby raises hopes that the survival rates in advanced or recurrent gastric cancer may thus increase with this treatment.

S-1 is active, even against disseminated peritoneal metastases in gastric carcinoma patients, as confirmed by Mori et al. (15) in a mouse model of gastric cancer with disseminated peritoneal disease. A high concentration of 5-FU has been detected in the intraperitoneal tumor lesions of the S-1 group, and prolonged survival rates were observed. However, the mechanism by which the high concentration of 5-FU were maintained in the peritoneal cavity is not yet known.

In more than 4,000 patients administered S-1 for gastric cancer, 25% have been reported to experience grade 3 or higher toxicities, and their median survival rate was 8.3 months (16). We herein observed an occurrence of grade 3 or higher toxicities in 9.4%, a response rate of 38.4%, and a median survival rate of 343 days in the patients who received S-1 monotherapy for advanced or recurrent gastric cancer (17).

In a report comparing S-1 plus CDDP administration and the single administration of S-1, side-effects with at least grade 3 severity were 43.5% and 22.5%, respectively, for each administration, and in combined therapy, side-effects occurred about twice as often as in single therapy, with 36.8% and 25.9% of RR, respectively, which is obviously higher in the combined therapy. However, MST was 319 days and 322 days, respectively, which indicates that the therapies did not contribute in any way to the extension of survival time (18). Therefore, when chemotherapy is started with S-1 alone and it is found to be ineffective, it is expected that adding CDDP to the therapy does not shorten the survival time but, instead, it maintains the response rate with less incidence of side-effects. Thus, we believe that our first-line and second-line therapies are reasonable. Similar to previous reports regarding side-effects with at least grade 3 in severity, non-hematological toxicity accounted for 10.5% and 46.2%, respectively, in S-1 and S-1 + CDDP, and hematological toxicity was 10.5% and 23.1%, respectively. Even when S-1 + CDDP was administered after S-1, side-effects did not increase significantly. S-1 + CDDP administration was performed 2.8 times on average, with a median value of 2 times, which is lower than the median value of 4 times in the previous report (11). This is due to the progression of the disease rather than side-effects. The response rate in the single administration of S-1 was 33.3% as expected, but the response rate in S-1 + CDDP in the second-line was 12.5%—far less than the 74% discovered by Koizumi et al. (11). It is believed that this is the reason for the decrease in the number of administrations of S-1 + CDDP.

We adopted this approach because paclitaxel used in this regimen showed no cross-resistance and had completely different side-effect profile (19).

At the beginning of this therapy, weekly paclitaxel therapy for gastric cancer had not been established, so we decided on a dose by referring to the reports for other types of cancer and tumors. In ovary cancers, 80 mg/m^2^ is the maximum-tolerable dose, but it has been reported that the incidence of neuropathy is frequently found with 60 mg/m^2^ (20). In breast cancers, it has been reported that C_max_ was high at 60 mg/m^2^ (21), which is recommended together with radiation therapy in lung cancers (22). From these reports, we decided to perform weekly paclitaxel therapy with 60 mg/m^2^ as the next chemotherapy for S-1 + CDDP. In recent reports of weekly paclitaxel therapy for gastric cancers, the dose of paclitaxel is 80 mg/m^2^, and we also provided weekly paclitaxel therapy as the second-line chemotherapy, following S-1 in pre-treatment, with a dose of 80 mg/m^2^. In this therapy, the RR was 0%, MST was 495 days, and 11.8% hematological toxicity with at least grade 3 in severity was recognized (23). In other reports of weekly paclitaxel therapy with a dose of 80 mg/m^2^, it was indicated that the initial treatment RR was 14.3%, MST was 221 days, side-effects with at least grade 3 in severity were 0%, and the median value of the number of times it was performed was 2.0 (24). In addition, it was reported that the dose was 70 mg/m^2^ in mixed cases with pretreatment and initial treatment, but the RR was 33%, MST was 160 days, hematological toxicity with at least grade 3 in severity was 27.2%, and the average number of times it was performed was 5.4 (25). In this therapy, the dose of paclitaxel was 60 mg/m^2^, and after treatment with S-1 and S-1 plus CDDP, neither CR nor PR developed. However, non-hematological toxicity was not found, and only 23.1% hematological toxicity was found. The average number of times it was performed was 3.8, with a median value of 4. As the third-line, following S-1 and S-1 plus cisplatin—protocols with a relatively high frequency of side-effects—a dose of 80 mg/m^2^ may be possible, but this is unclear, as we do not have any relevant experiences.

Thus, in this therapy, the response rate was low, but survival time was excellent. Average survival time was about 1,000 days, and no reports with outcomes too good to calculate MST were found in our research. We believe that this was caused by the smooth switch of regimens. It is difficult to switch from one type of chemotherapy to another regimen if there is no obvious PD, but even in cases of peritoneal dissemination in which PD is difficult to determine, we switched regimens with increased tumor markers and palpation of the Douglas cavity. Therefore, we were able to switch to the next regimen without any hesitation, because the next regimen had already been determined.

In addition, as shown in Figure 3, it was concluded that resection of a primary tumor can further improve outcomes, even when it is not a curative resection. However, no reports clearly indicating this have been found.

In conclusion, this report is of the results from the first-line chemotherapy with S-1 alone, second-line with S-1 plus cisplatin, and third-line fixed with weekly paclitaxel. In this therapy, direct curative effects for tumors were not high, but good survival rates were seen, and the side-effects were minor.

## Figures and Tables

**Figure 1:**
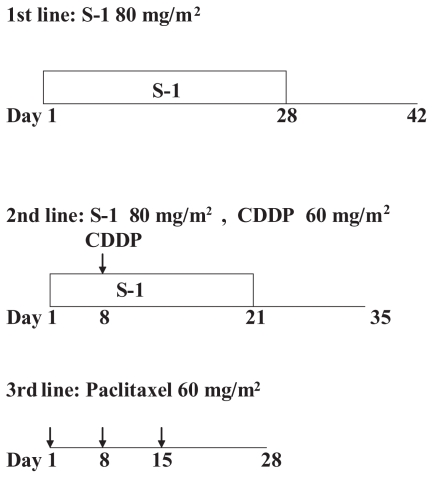
Protocols of First-, Second-, and Third-line chemotherapy **First line chemotherapy:** S-1 was administered orally twice daily after breakfast or dinner, at 80 mg/m^2^/day for 4 weeks, followed by 2 weeks of rest. **Second line chemotherapy:** S-1 was administered at the same dosage as the first line chemotherapy. S-1 was administered for 3 weeks followed by 2 weeks of rest. On day 8, S-1 was combined with cisplatin at 60 mg/m^2^. **Third line chemotherapy:** Paclitaxel was administered at 60 mg/m^2^ on day 1, 8, and 15 of a 4-week treatment cycle.

**Table 1:** Characteristics of enrolled patients.

Characteristcs	Number of patients	(%)
Total number of patients	19	
Age (years)		
Mean ± SD (range)	61.3 ± 1.4(38–75)	
Sex		
Male	13	68.4
Female	6	31.6
Karnofsky performance status		
80–100	19	100
Histological type (Japanese classification)		
Differntiated	5	26.3
Undifferentiated	14	73.7
Primary treatment		
Curative gastrectomy	6	31.6
Palliative gastrectomy	10	52.6
Without gastrectomy	3	15.8
Target lesions		
primary	3	15.8
peritoneal dissemination	14	73.3
lymph node metastasis	5	26.3
liver metastasis	2	10.5

**Table 2:** Adverse events, according to regimen group.

Regimen	Type	Grade				
		**1**	**2**	**3**	**4**	**%3 and 4**
**TS1 (n** = **19)**						
	diarrhea	2	3	0	0	0
	constipation	1	0	0	0	0
	nausea	2	1	0	0	0
	anorexia	1	5	0	0	0
	vomiting	1	1	1	0	5.3
	headache	2	0	0	0	0
	fatigue	7	2	0	0	0
	stomatitis	0	5	1	0	5.3
	dizziness	1	1	0	0	0
	AST,ALT	2	1	0	0	0
	lachrymation	3	1	0	0	0
	photophobia	3	0	1	0	5.3
	dysgeusia	1	1	0	0	0
	alopecia	1	0	0	0	0
	pigmentation	3	1	0	0	0
	nail changes	1	1	0	0	0
	conjunctivitis	1	0	0	0	0
	numbness of limbs	2	0	1	0	5.3
	abdominal pain	1	0	0	0	0
	edema	1	1	0	0	0
	allergic rhinitis	1	1	0	0	0
	anemia	0	1	0	0	0
	leukopenia	0	1	1	0	5.3
	neutropenia	0	0	1	0	5.3
**TS1** + **CDDP (n** = **13)**						
	diarrhea	2	0	2	0	15.4
	nausea	0	0	1	0	7.7
	anorexia	2	1	4	0	30.8
	fatigue	2	2	1	0	0
	AST,ALT	0	1	0	0	0
	alopecia	1	0	0	0	0
	pigmentation	2	0	0	0	0
	abdominal pain	0	1	1	0	7.7
	edema	0	1	0	0	0
	hypotension	1	0	0	0	0
	epistaxis	1	0	0	0	0
	rash	1	0	0	0	0
	anemia	0	1	2	0	15.4
	leukopenia	0	1	3	1	30.8
	neutropenia	0	0	2	0	15.4
	lymphopenia	0	0	0	1	7.7
**Weekly paclitaxel (n** = **13)**						
	diarrhea	2	1	0	0	0
	constipation	0	1	0	0	0
	nausea	3	0	0	0	0
	anorexia	1	0	0	0	0
	fatigue	3	0	0	0	0
	dizziness	0	1	0	0	0
	AST,ALT	1	1	0	0	0
	lachrymation	1	0	0	0	0
	dysgeusia	1	0	0	0	0
	alopecia	3	1	0	0	0
	numbness of limbs	2	1	0	0	0
	edema	0	1	0	0	0
	insomnia	1	0	0	0	0
	anemia	0	0	1	0	7.7
	leukopenia	0	1	2	0	15.4
	neutropenia	0	1	2	0	15.4
